# Intraoral Schwannoma Presenting as a Palatal Mass in a Pediatric Patient

**DOI:** 10.1002/ccr3.71598

**Published:** 2025-12-03

**Authors:** Saede Atarbashi‐Moghadam, Ali Lotfi, Mohammadreza Kashefi Baher

**Affiliations:** ^1^ Department of Oral and Maxillofacial Pathology, School of Dentistry Shahid Beheshti University of Medical Sciences Tehran Iran; ^2^ Health Research Center, Chamran Hospital Tehran Iran

**Keywords:** neurilemmoma, Oral cavity, palate, schwannoma

## Abstract

Schwannomas, though rare in the pediatric oral cavity, can present as painless palatal swellings that mimic other benign lesions. This case highlights the importance of including schwannoma in the differential diagnosis of palatal masses in children to ensure early recognition and appropriate surgical management.

## 1 Case Presentation

**FIGURE 1:**
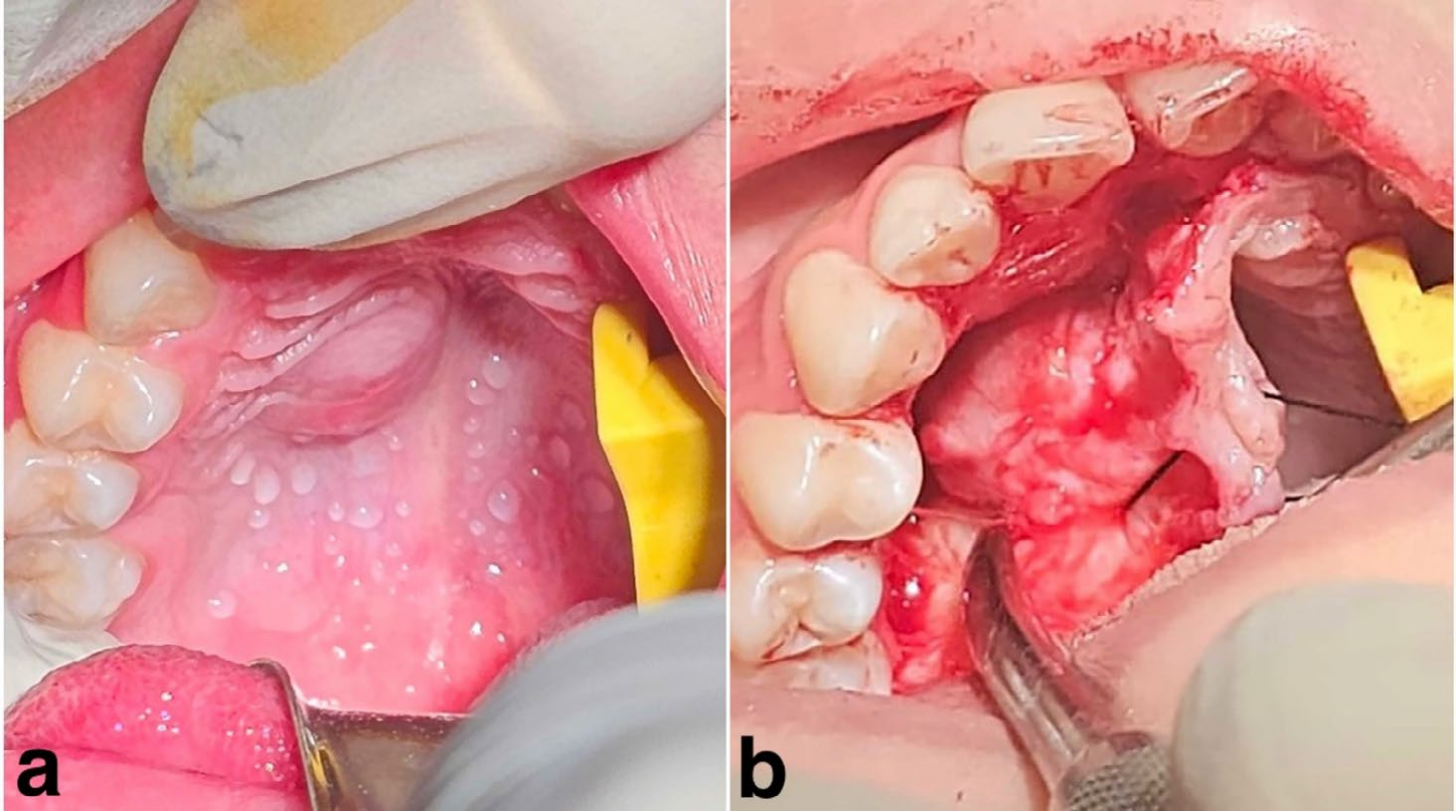
(a) Solitary, well‐circumscribed submucosal mass in the right hard palate. (b) The lesion is being exposed at the time of surgery.

A 12‐year‐old girl was referred to a private oral pathology center with an 18‐month history of painless palatal swelling. Intraoral examination revealed a well‐circumscribed submucosal mass in the right hard palate, measuring 2.0 × 1.8 cm. The lesion matched the surrounding mucosa in color, had a soft to fluctuant consistency, and showed no signs of surface erosion or ulceration (Figure 1a). The panoramic radiograph revealed no intraosseous lesion, and the adjacent teeth responded positively to pulp testing. Extraoral examination demonstrated no abnormalities, and laboratory findings were within normal limits. Excisional biopsy was performed under general anesthesia to establish a definitive diagnosis, revealing a peripheral lesion with intact adjacent bone (Figure 1b).

**FIGURE 2:**
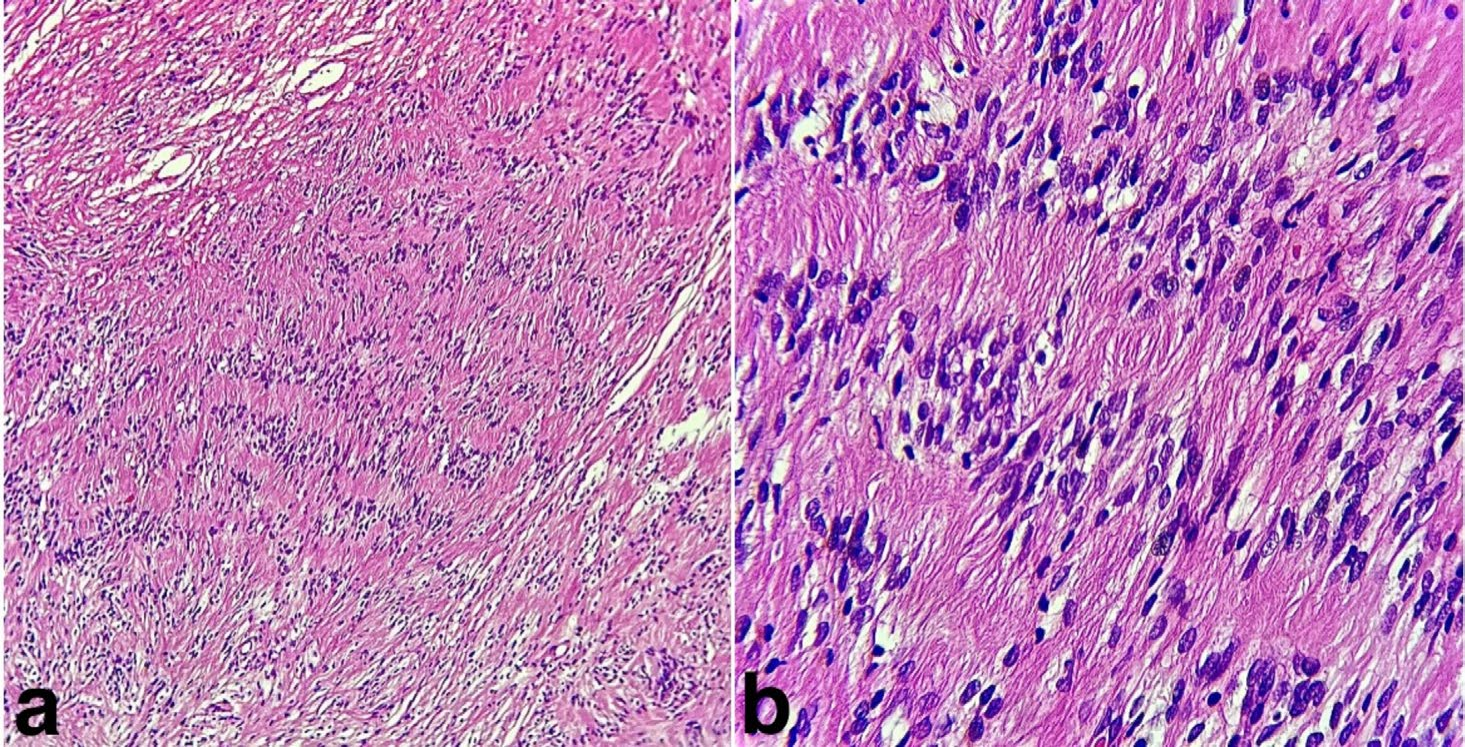
(a) Streaming fascicles of spindle‐shaped cells forming well‐organized Antoni A tissue (H & E × 100). (b) Verocay bodies (H & E × 400).

**FIGURE 3:**
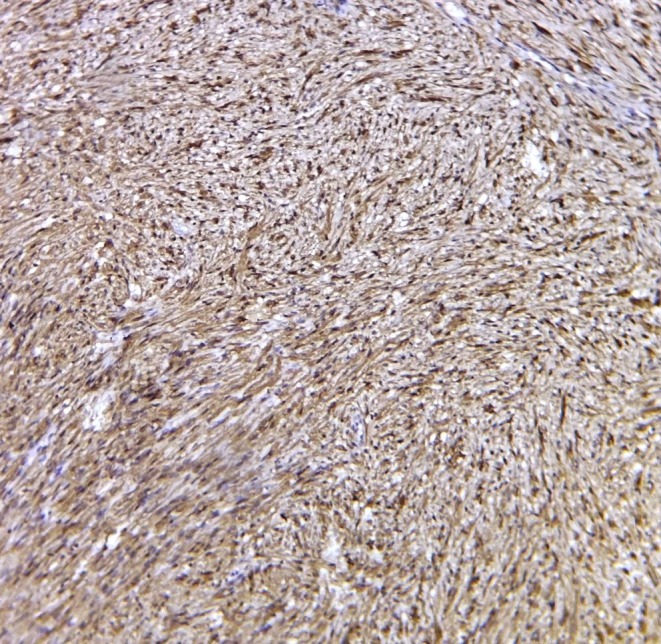
Diffuse expression of S‐100 protein in the cytoplasm of schwann cells as brown coloration (IHC × 100).

Microscopy revealed an encapsulated neural tumor composed of streaming fascicles of spindle‐shaped cells forming well‐organized Antoni A tissue (Figure 2a) with Verocay bodies (Figure 2b), along with less organized Antoni B regions, findings consistent with schwannoma. Moreover, immunohistochemical (IHC) analysis revealed diffuse cytoplasmic S‐100 protein expression in Schwann cells (Figure 3). The patient is currently under follow‐up at 3 months.

## 2 Discussion

Schwannomas (neurilemmomas) are benign neoplasms arising from the myelin sheath, with unclear etiology [1]. Schwannomas typically arise in the third to fifth decades of life with no gender predilection [2]. However, this case presented at an unusually early age.

Neural lesions are uncommon in the oral cavity [2]. However, schwannomas often arise in the head and neck (H&N) region, most frequently along the course of the vestibulocochlear nerve [1]. Moreover, H&N schwannomas account for 25%–48% of all extracranial cases, yet only 0.2%–1% occur in the oral cavity, predominantly involving the tongue [1, 3].

Clinically, these lesions typically manifest as painless, solitary nodules with slow longitudinal growth along the nerve, producing fusiform enlargement while preserving neural integrity. The presence of pain or neurological deficits may raise concern for malignant transformation [2, 3].

Schwannomas usually lack distinctive clinical features and may be detected incidentally, with definitive diagnosis relying on histopathological evaluation [2]. Herein, an inflammatory periapical lesion was first ruled out because of the absence of radiographic evidence and the vitality of the adjacent teeth. The diagnostic hypotheses following this exclusion included benign salivary gland tumors (e.g., pleomorphic adenoma) and benign mesenchymal tumors (e.g., neurofibroma, palisaded encapsulated neuroma, lipoma, hemangioma). Odontogenic tumors were excluded owing to their predominantly intraosseous presentation. In this case, if the lesion were located at the anterior midline, a nasopalatine duct cyst would also warrant consideration.

IHC markers such as S‐100, SOX‐10, and calretinin may aid diagnosis but are not mandatory [2]. IHC investigation in this case confirms the neural origin of the cells.

Surgical excision is the treatment of choice, and recurrence is rare [1].

## Author Contributions

**Saede Atarbashi‐Moghadam:** conceptualization, writing – original draft, writing – review and editing. **Ali Lotfi:** data curation, formal analysis, validation. **Mohammadreza Kashefi Baher:** supervision, writing – original draft, writing – review and editing.

## Funding

The authors have nothing to report.

## Ethics Statement

The authors have nothing to report.

## Consent

Written informed consent for the publication of this case, including all clinical details and any accompanying images, was obtained from the patient's parents.

## Conflicts of Interest

The authors declare no conflicts of interest.

## Data Availability

No datasets were generated or analyzed during this study; therefore, data sharing is not applicable.

## References

[1] R. H. Phulware , R. Sardana , D. S. Chauhan , A. Ahuja , and M. Bhardwaj , “Extracranial Schwannomas of the Head and Neck: A Literature Review and Audit of Diagnosed Cases Over a Period of Eight Years,” Head and Neck Pathology 16, no. 3 (2022): 707–715. 35157211 10.1007/s12105-022-01415-y PMC9424433

[2] V. Dokania , A. Rajguru , V. Mayashankar , I. Mukherjee , B. Jaipuria , and D. Shere , “Palatal Schwannoma: An Analysis of 45 Literature Reports and of an Illustrative Case,” International Archives of Otorhinolaryngology 23, no. 3 (2019): 360–370. 10.1055/s-0039-1692635 PMC6660292 31360259

[3] Y. Harazono , K. Kayamori , J. Sakamoto , et al., “Retrospective Analysis of Schwannoma in the Oral and Maxillofacial Region: Clinicopathological Characteristics and Specific Pathology of Ancient Change,” British Journal of Oral & Maxillofacial Surgery 60, no. 3 (2022): 326–331. 34690015 10.1016/j.bjoms.2021.07.014

